# The impact of 17β-estradiol on the estrogen-deficient female brain: from mechanisms to therapy with hot flushes as target symptoms

**DOI:** 10.3389/fendo.2023.1310432

**Published:** 2024-01-08

**Authors:** Katalin Prokai-Tatrai, Laszlo Prokai

**Affiliations:** Department of Pharmacology & Neuroscience, University of North Texas Health Science Center, Fort Worth, TX, United States

**Keywords:** aromatase, DHED, 17β-estradiol, hot flush, hypoestrogenemia, menopause, sex steroid, vasomotor symptoms

## Abstract

Sex steroids are essential for whole body development and functions. Among these steroids, 17β-estradiol (E2) has been known as the principal “female” hormone. However, E2’s actions are not restricted to reproduction, as it plays a myriad of important roles throughout the body including the brain. In fact, this hormone also has profound effects on the female brain throughout the life span. The brain receives this gonadal hormone from the circulation, and local formation of E2 from testosterone *via* aromatase has been shown. Therefore, the brain appears to be not only a target but also a producer of this steroid. The beneficial broad actions of the hormone in the brain are the end result of well-orchestrated delayed genomic and rapid non-genomic responses. A drastic and steady decline in circulating E2 in a female occurs naturally over an extended period of time starting with the perimenopausal transition, as ovarian functions are gradually declining until the complete cessation of the menstrual cycle. The waning of endogenous E2 in the blood leads to an estrogen-deficient brain. This adversely impacts neural and behavioral functions and may lead to a constellation of maladies such as vasomotor symptoms with varying severity among women and, also, over time within an individual. Vasomotor symptoms triggered apparently by estrogen deficiency are related to abnormal changes in the hypothalamus particularly involving its preoptic and anterior areas. However, conventional hormone therapies to “re-estrogenize” the brain carry risks due to multiple confounding factors including unwanted hormonal exposure of the periphery. In this review, we focus on hot flushes as the archetypic manifestation of estrogen deprivation in the brain. Beyond our current mechanistic understanding of the symptoms, we highlight the arduous process and various obstacles of developing effective and safe therapies for hot flushes using E2. We discuss our preclinical efforts to constrain E2’s beneficial actions to the brain by the DHED prodrug our laboratory developed to treat maladies associated with the hypoestrogenic brain.

## 1 Introduction

The brain abundantly expresses receptors to many hormones, including steroids hormones (1–3). One of the common elements of these hormones is that all of them are synthesized from cholesterol with the aid of an enzyme machinery through a multistep biosynthetic pathway called steroidogenesis (4, 5). Therefore, these hormones carry a 17-carbon skeleton built from four rings that are characteristically fused together as in cyclopentanoperhydrophenanthrene.

**Figure 1:**
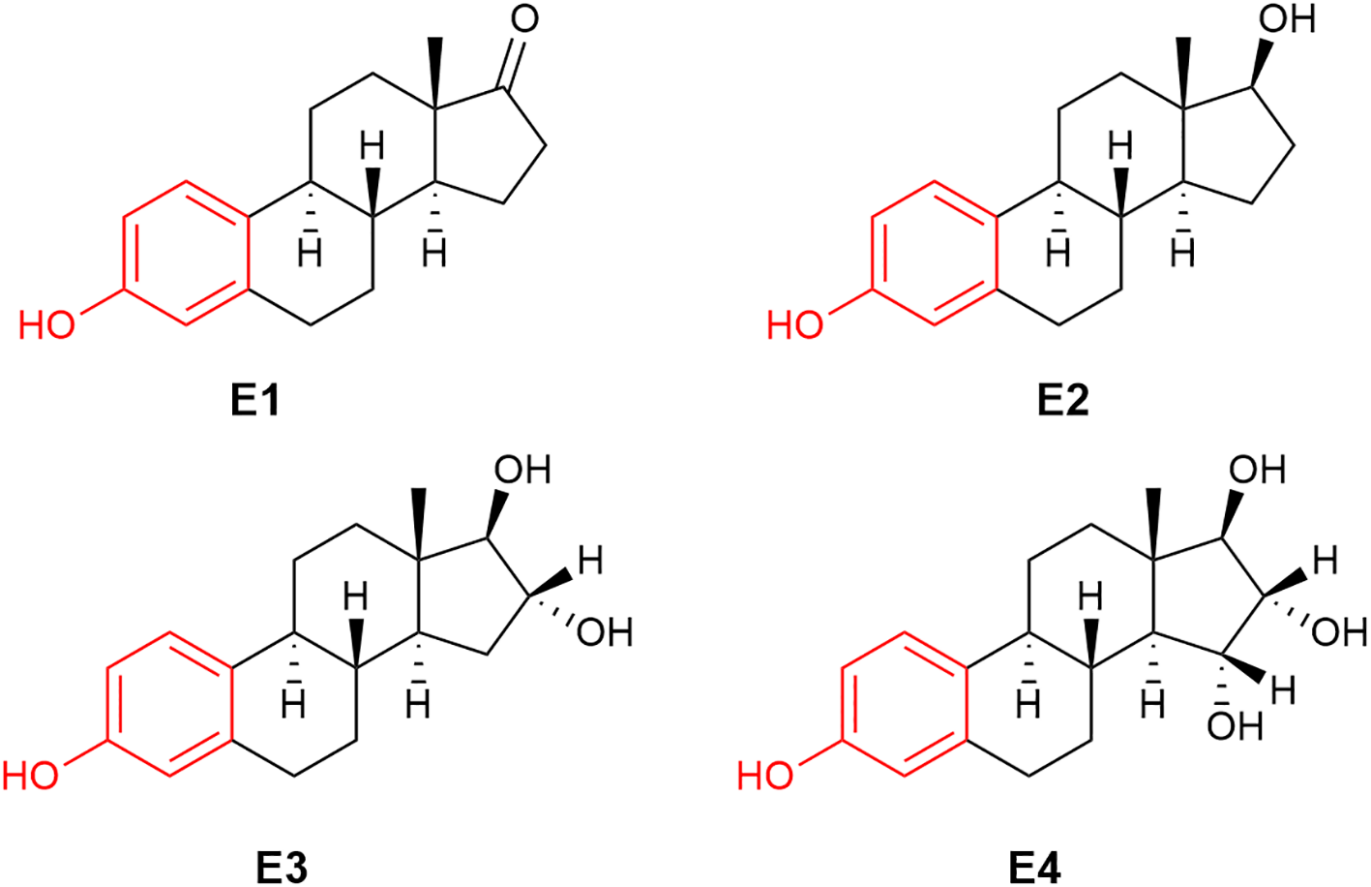
The chemical structure of human estrogens: estrone (E1), 17β-estradiol (E2), estriol (E3) and estetrol (E4).

Among steroid hormones, estrogens, androgens, and progestogens are also called sex hormones owing to their critical contributions to reproduction, sexual differentiation, and development. Beyond these characteristic roles, these distinct hormones also have a myriad of effects and functions throughout the entire body in both sexes, although in a sexually dimorphic manner (2, 6–10). Androgens such as testosterone (T) have also been called “male hormones” due to their masculinizing effects (11). On the other hand, estrogens such as E2 (**Figure 1**) are commonly known as “female hormones.” Progesterone (P4) is the other main “female” sex hormone associated with fertility and pregnancy. It is an endogenous progestogen, whereas progestins are synthetic progestogens commonly used in birth control pills and hormone replacement therapies by women with intact uterus (12, 13).

Peripheral organs, primarily the adrenal glands and the gonads, secret sex steroid hormones in both sexes, and these lipophilic “neuroactive” hormones reach the brain from the circulation because they are capable of diffusing through the blood-brain barrier (7, 14–16). Additionally, they are also synthesized locally irrespective of sex, for example, in the brain (and in the central nervous system in general)—either from blood-borne precursors or *de novo* from cholesterol (5, 6). Therefore, the brain is not only a target for but also a maker of these steroids. The centrally formed hormones are distinguished with the term “neurosteroids” (17, 18).

Sex hormones’ diverse regulatory roles within the nervous system are governed by genomic and non-genomic effects (6, 19–21). The classical genomic pathway involves full transcriptional activities through the involvements of their respective nuclear receptors—the progesterone, estrogen, and androgen receptors. These receptors exhibit a common modular domain structure that consists of the C-terminal ligand-binding and N-terminal domains, as well as the central DNA-binding domain (22). These receptors are abundantly yet unevenly distributed in the brain. For example, a large density of sex steroid receptors is localized in the hippocampus (10). Additionally, and importantly, rapid extra-nuclear, non-genomic cellular responses are also exerted by these steroids without gene transcription. In these scenarios, regulations of gene expressions could occur *via* signaling cascades through interactions with various membrane-bound receptors and putative receptors, as well as through the regulation of ion channels among others (23–25). These two distinct mechanisms can also influence each other to regulate associated physiological responses (26).

Extensive research during the last several decades has revealed that sex hormones control brain and behavioral trajectories across the life span (2, 27–29). Sex differences in the developing and adult brain have been recognized (30–34). These differences are attributed, at least in part, to exposure to sex steroids during the embryonic state and throughout development, which results in regional brain organization differences, ultimately leading to different behavioral, cognitive, emotional, and adoptive outcomes between the sexes, as well as among age groups within the same sex (10, 35–37).

The “female” sex hormones E2 and progesterone are essential for brain health and well-being across women’s life spans. Here, we specifically focus on E2’s impacts on the hypoestrogenic or estrogen-deficient female brain using hot flushes as typical vasomotor symptoms (VMS). The latter affect most women at midlife and can last up to 10 years and longer, often adversely impacting the health-related quality of life (38–40).

## 2 A brief overview of human estrogens

The term “estrogen” is derived from *oistros* and *oestrus* (Greek and Latin for gadfly) as a reference to estrus (mating) stimulatory effect of this type of compounds (41). Very frequently, the term “estrogen” is used for the most well-known human estrogen, E2 (**Figure 1**). It should be noted that there are three additional estrogens produced in females at various stages of their life spans: these are estrone (E1), estriol (E3) and estetrol (E4), whose chemical structures are also shown in **Figure 1**. While E2 is the most prominent estrogen in females prior to menopause, E1 becomes the principal estrogen in postmenopausal women (42). E1 can also be reversibly metabolized to E2 by 17β-hydroxysteroid dehydrogenase. E3 and E4 are fetal estrogens that are present only during gestation (43). E4 also is the estrogenic constituent of a recently approved combination oral contraceptive marketed as Nextstellis^®^ (44).

These estrogens and other vertebrate estrogens such as 17α-estradiol, as well as a diverse class of estrogenic compounds bind to their cognate nuclear estrogen receptors (ERs) ERα and ERβ with various affinities (45). E2 is the most potent estrogen in vertebrates; therefore, it is our ER-ligand of choice highlighted in this review. ERs are richly expressed throughout the body including the brain where they are prominently present, for example, in the hippocampus, prefrontal cortex and hypothalamus (46, 47). Therefore, it is not surprising that E2’s effects on the brain involves the classical genomic mechanisms (48). Briefly, like other members of the nuclear receptor superfamily of proteins (22), ERs produce their genomic effect through gene transcription driven by E2’s ligation to ERα and ERβ. After E2 is distributed into a cell and reached the nucleus, it binds to ERs. Then, ligated ERs assemble to homo- or heterodimers that bind to the estrogen-response element (ERE) of the nuclear DNA (49). Gene transcription is enabled by the amino-terminal transactivation domain and the carboxy-terminal ligand-binding domain of the ERs (50). The assembly of ERs with co-regulators/co-repressors is highly dependent on posttranslational modifications and epigenetic regulation. On the other hand, ERE-independent mechanisms of estrogen action have also been shown (51). In addition, several isoforms of ERα and ERβ arise through alternative splicing, many of which alter estrogen-mediated gene expression (49). Combined with the dependence on the receptor isoform, ligand, promoter, cell type, and intricate epigenetic regulation of the genomics pathways (49, 52), our understanding of the hormone’s action in the brain by genomic mechanisms is nevertheless incomplete, especially in the context of VMS. However, effective treatment of hot flushes is achieved primarily through activation of ERα with estrogens but is also associated with increased risk for breast and uterine cancer (53). Extranuclear ERs are also present as membrane-associated homo- or heterodimers at the cell membrane and in the cytoplasm (49, 54). In addition, a G protein-coupled ER (GPER) is found in intracellular membranes, and its activation results in intracellular Ca^2+^-mobilization and synthesis of phosphatidylinositol 3,4,5-triphosphate in the cell’s nucleus (55). This signaling mechanism can also regulate gene transcriptions. For example, the mitogen-activated protein kinase cascade and the cyclic-AMP-responsive element-binding protein signaling pathway respond rapidly to E2 and have been implicated in the hormone’s effects in the brain (56, 57). The estrogen-ER complex was also shown to signal in the cytoplasm (58).

**Figure 2:**
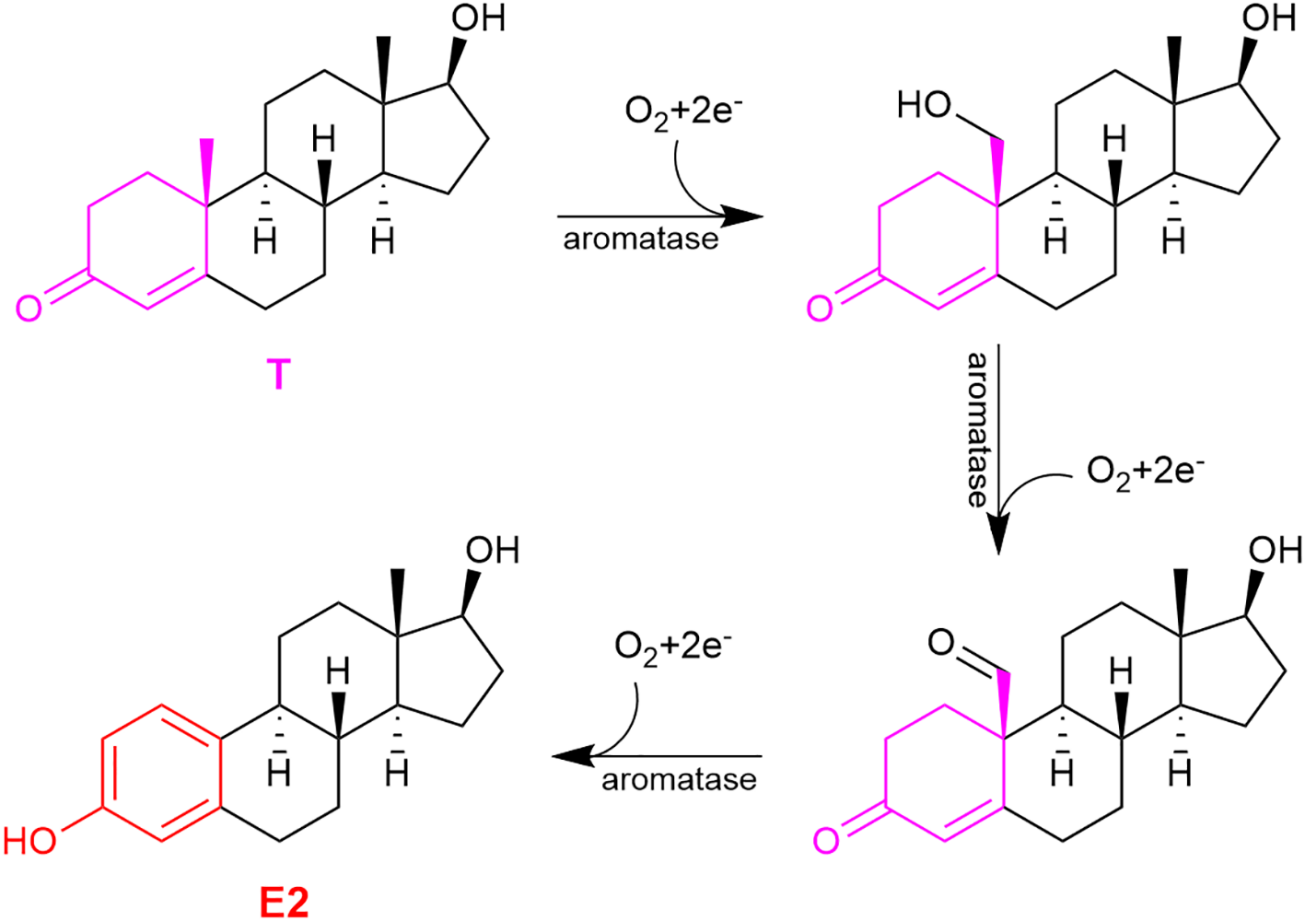
The three-step oxidation of testosterone (T) to 17β-estradiol (E2), catalyzed by aromatase.

Estrogens’ chemical structures are distinct among sex steroids, because they are the only steroids that possess a phenolic A-ring (**Figure 1**, red ring). During E2’s sequential formation from cholesterol, T (or androstenedione for E1) produced in the penultimate step undergoes a three-step oxidation of the methylcyclohexenone (moiety in magenta) to the phenolic A-ring (**Figure 2**). These reactions are catalyzed by aromatase also known as estrogen synthase (48, 59). This enzyme is found in many gonadal and extragonadal sites throughout the body and regardless of sex (5, 60–62). In females, the ovary is the principle gonadal source of circulating E2 before menopause. After menopause, the expression of aromatase in adipose tissues is significantly increased (42).

The phenolic A-ring also permits estrogens to act as powerful direct free-radical scavenging antioxidants, which is critical to tackle oxidative stress implicated in the initiation and progression of neurodegeneration (63). Although it is beyond the scope of this review to address neuroprotection per se, the potent broad-spectrum neuroprotective action of E2 should be recognized. This is owing to the well-orchestrated genomic and non-genomic actions by the hormone, which allows to collectively thwart both the initiation and progression of neuronal cell death (48).

## 3 E2 and the aging female brain

The brain also is an important extragonadal site of aromatase expression; hence, this organ is capable of producing E2—most prominently in the hypothalamus, as well as in the hippocampus, cerebral cortex, cerebellum, and brainstem (64). It should be noted that most information in this regard are coming from animal studies. An interesting and perhaps unexpected notion in this regard is that the locally formed E2 causes masculinization in the embryonic rodent brain (65, 66). However, hormone-mediated differences observed during development and involving aromatase are transient, and functional consequences of feminized versus masculinized traits on the adult brain are not yet known (67). Moreover, androgens (i.e., not estrogens) appear to be the principal masculinizing hormones in non-human primates and humans (66). Nevertheless, the abundant central distribution of this enzyme implies a pleiotropic, yet perhaps on demand only role of the locally synthesized E2 in the brain. On cellular level, brain aromatase is mainly expressed in neurons. However, animal studies have revealed the expression of this enzyme in astrocytes and glia, especially following excitotoxic injury or trauma (68, 69).

Multiple lines of evidence show that E2 directly affects structural and, with this, functional and behavioral trajectories in the brain throughout the lifetime of a female (9, 27, 70–72). However, the available endogenous circulating E2 supply is not steady across the lifespan but varies not only with chronologic age but also within the reproductive period, as it fluctuates with the menstrual cycle, significantly increases during pregnancy, and then drops postpartum (73–75). Moreover, a substantial portion of the reproductive-age population uses oral contraceptives, which also exposes them to exogenous estrogen (76). This further complicates the already complex situation owing to multiple confounding factors acting in concert (e.g., genetic variations, age, diet, comorbidity status) to delineate the direct impact of E2 on the female brain.

A drastic and steady decline in circulating E2 occurs naturally over an extended period of time starting with the perimenopausal transition, as ovarian functions are gradually declining until the complete cessation of the menstrual cycle due to anovulation (38, 72). During the transition period to full menopause and geripause, the body including the circuits of the brain must somehow adjust to the changing estrogenic milieu and eventually compensate for or adapt to hypoestrogenemia. It has become evident that a continual E2-deficient state recognized as post-menopause broadly and adversely impacts neural and behavioral functions especially those associated with the prefrontal cortex, hippocampus, and hypothalamus (9, 19, 27, 70–72). This may lead to or exacerbate a constellation of symptoms such as cognitive frailty, mood and sleep disturbances, vasomotor symptoms (VMS), as well as susceptibility to develop age-related neurodegenerative diseases, among them Alzheimer’s disease—just to mention a few unpleasant consequences of the hypoestrogenic or estrogen-deficient aging female brain (39, 40, 74, 77, 78).

It remains unclear, however, how the local *de novo* synthesis of E2 is operating at this stage in the female human brain. Apparently, the locally synthesized E2 (at least in certain brain regions) is not enough to preserve an “estrogenic” brain and, thus, to ameliorate menopause-associated symptoms, although genetic factors may be very important in this regard. A recent study reported that an active aromatization process takes place in post-menopausal women after an acute ischemic stroke (79). However, this finding pertains to the periphery only and does not provide evidence on brain aromatase activity in an injured brain. In all cases, many additional studies will be needed to understand the role and function of the aromatase, as well as ERs’ functions in the aging female brain.

Alongside, the adverse consequences of age-associated decline of circulating E2 strongly implies that the hormone per se also is a therapeutic molecule (although with some limitations) to “re-estrogenize” the brain and lessen menopause-/age-associated functional declines and maladies (59). In this regard, the controversial Women's Health Initiative (WHI) studies should be noted, which perpetuated, regrettably, the “all estrogens are created equal” dogma. Conjugated equine estrogens (CEE) extracted from pregnant mare urine alone (Premarin^®^) or together with the synthetic progestin medroxyprogesterone acetate (Prempro^®^) were used in this large-scale yet abruptly halted trial instead of “hormone replacement” that would have required the use of human hormones depleted with aging (80–82). A greater risk of developing invasive breast cancer and thromboembolic stroke was observed in WHI trials, in part due to the significant peripheral exposure to CEE, prompting many women to stop taking this type of medications yet leaving them frustrated because of a dearth of effective treatment options to preserve a high quality of life involving their mental health and well-being.

Decline of many brain functions is prevalent upon aging and menopause, which is believed to be closely related to the waning of endogenous E2. Therefore, estrogen therapy (ET) should be beneficial in this regard. However, conventional ET carries risks, including but not limited to cardiovascular liabilities as well as enhanced chances for the development of breast and uterine cancers, especially if it is used around geripause (80–83). Timing of ET is one of the critical factors. In fact, a recent review (84) analyzing over 3500 publications in the field concluded that the “critical age window period” was one of the most addressed topics in ET-related publications in the last 10 years focusing on cognition, one of the most studied higher-order brain functions in the present context.

Various neuroimaging techniques can reveal important information on how the female brain structure and connectivity change with aging and respond to exogenous E2, for example, in the context of hippocampal functions. These state-of-the-art approaches are not widely accessible to the scientific community for a variety of reasons, including but not limited to ethical and financial considerations (8, 85, 86). Therefore, animal studies especially involving affordable rodent models are invaluable, although they are not without limitations (87). Yet, these paradigms allow for untangling how E2 deprivation affects under a controlled endocrine environment and, in turn, how E2 treatment modulates various brain regions with consequential functional and behavioral outcomes. Classical example for this is using ovariectomized rodents to eliminate estrus cycle-related confounds and, thus, to simulate ovarian failure in females. Additionally, 4-vinylcyclohexene diepoxide treatment has been shown to lead to ovarian follicle loss over time, mimicking thereby more accurately the progressive human menopausal transitions (88).

Below we focus on VMS as archetypic manifestation of E2 deficiency of the aging brain and discuss current mechanistic understanding of this symptom. We also examine proven and promising recent therapeutic remedies for hot flushes that negatively impact the mental well-being of menopausal women. In this context, we highlight a preclinical effort our laboratory has championed to concentrate the therapeutic E2 only to the brain for a potential development of an inherently safe and efficacious ET to tackle VMS.

## 4 E2 and VMS (hot flushes)

In the next 20–25 years, an estimated 1 billion women worldwide will be older than 50 years and, therefore, experience perimenopausal or menopausal symptoms (38, 72, 89). VMS are commonly accompanying these periods manifesting hot flushes and/or night sweats with varying severity among women and, also, over time within an individual. It is estimated that around three-fourths of women experience midlife hot flushes now considered the hallmark symptom of menopause (39, 40, 90, 91). Women with frequent VMS also experience higher rates of depression, anxiety, and sleep disturbances lowering their quality of life. Therefore, efficacious, and safe pharmacological interventions are critical, although the therapeutic regimen (medication of choice, dose, dosage form, route of administration, time, and duration of treatment) should be individualized.

### 4.1 Mechanism of VMS

The physiology of VMS is not completely understood, although our understanding in this regard is continuously “heating up.” VMS appears to be related to abnormal changes in the thermoregulatory center of the body; viz., the hypothalamus particularly involving its preoptic and anterior areas. This leads to altered vasodilatory response to even slight elevations of core body temperature (92–94). Hot flushes start with the gradual decline or unpredictable fluctuation in circulating E2 during perimenopause. Past menopause, the body apparently adapts to the hypoestrogenic state, as hot flushes fade away in most women. Accordingly, perhaps there is a critical “window of opportunity” in this regard for the better understanding of the process leading to hot flushes. Because of the strong association between VMS and declining E2, systemic hormone therapy seems to be the most effective treatment against moderate-to-intense hot flushes (93, 94). Deficiency of E2, however, may not be the sole reason for developing hot flushes, although it is most probably the trigger event.

Neurotransmitters, particularly serotonin and norepinephrine, as well as the neuropeptide neurokinin B (NKB) have also been indicated as causative factors in hot flushes (95–97). Perhaps it is not surprising, because E2 modulates the synthesis and release of these neurotransmitters, as well as the expressions of their receptors, which in turn affect comprehensive thermoregulatory responses. Pharmacological activation of the receptors of NKB in neurons of the medial preoptic area of hypothalamus also elicits a robust decrease in core temperature and alters skin vasomotion (97).

Additionally, kisspeptin, NKB and dynorphin neurons project to hypothalamic structures and modulate the heat-defense pathway through NKB signaling (98). This recognition by the Rance group culminated in the very recent development and US Food and Drug Administration (FDA) approval of a neurokinin-3 receptor antagonist (Veozah^™^, fezolinetant) as a non-hormonal treatment against hot flushes. Interestingly though, one of the side-effects of this medication listed is hot flushes (99). Some of the heat-sensitive neurons in the preoptic area are GABAergic (γ-aminobutyric-acid-releasing) and their excitation in rats results in tail skin vasodilatation through projections that inhibit tonic activation of sympathetic (vasoconstrictor) preganglionic neurons in the ventromedial medulla (100). Therefore, activation of these GABAergic neurons by NKB has been linked to reduced vasomotor tone.

### 4.2 E2 and hot flushes

Although the neurotransmitters discussed above may be useful to target for a potential non-hormonal management of VMS (i.e., without involving estrogens), none of these approaches has been efficacious enough and/or without side effects thus far (95, 96, 101). ET remains the most effective against VMS probably due to the pleiotropic action of the hormone on warm-sensitive neurons to maintain in their synapses a neurotransmitter/neuromodulator composition that results in basal activity without triggering heat dissipation (40, 101). In the absence of E2 or an estrogen, the “abnormal” balance in neurotransmitters/neuromodulators of the synapses will cause activation of the descending heat dissipating pathways, accompanied by sweating, vasodilation, and subsequent heat loss manifesting as hot flushes. ET could prevent debilitating VMS by restoring a “healthy” balance of these neurotransmitters/neuromodulators in the neural circuits that maintain temperature homeostasis (102).

Notwithstanding, current ET carries contraindications due to the already mentioned risks for deep vein thrombosis and subsequent pulmonary embolism, estrogen-sensitive cancers, and additional adverse effects (40, 80, 82, 83). This is, in large part, due to the significant peripheral exposure to the hormone and, thus, a significant increase in circulating estrogen(s) even when E2 is used for intervention instead of CEE. Therefore, safe ET must be selectively and specifically confine E2’s beneficial actions into the brain when the therapeutic objective is to alleviate symptoms of central origin. Previous preclinical attempts in this regard brought about modest results without translational value (103).

**Figure 3:**
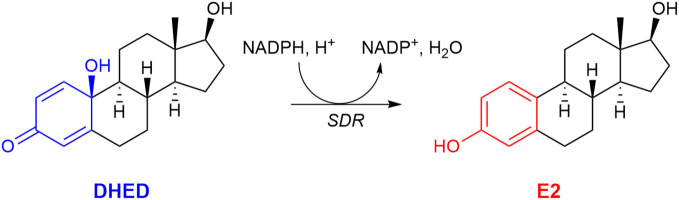
The bioprecursor prodrug 10β,17β-dihydroxyestra-1,4-dien-3-one (DHED) converts to 17β-estradiol (E2) in the brain by reduction catalyzed by a short-chain dehydrogenase/reductase (SDR) and relying on (NADPH) as a cosubstrate.

Our laboratory, however, was able to develop a unique prodrug approach to target E2 into the brain while sparing the rest of the body from unwanted off-target E2 exposure (59, 104). As shown in **Figure 3**, our bioprecursor prodrug 10β,17β-dihydroxyestra-1,4-dien-3-one (DHED) selectively converts to E2 after systemic administration but remains inert in the rest of the body. Therefore, DHED treatments efficiently alleviate symptoms that originate from the hypoestrogenic brain in numerous preclinical animal models of centrally regulated and estrogen-responsive maladies without peripheral hormonal liability (59). Specifically, circulating E2 levels measured by a validated bioassay using liquid chromatography–tandem mass spectrometry (105) showed no increase after DHED treatments compared to those of the untreated control animals. Additionally, lack of weight gain by estrogen-sensitive peripheral organs, such as the uterus and the anterior pituitary, as well as lack of MCF-7a breast cancer cell xenografts’ proliferation used as surrogate biomarkers have unequivocally shown the distinguishing feature of the DHED approach in terms of targeting E2 to the brain without its peripheral formation from the inert prodrug (59, 104).

**Figure 4:**
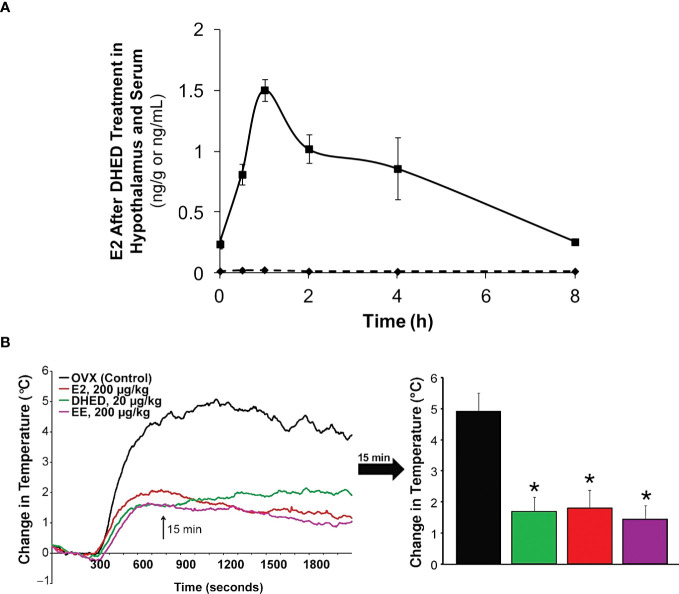
**(A)** After DHED treatment of ovariectomized (OVX) Sprague-Dawley rats (200 µg/kg, p.o.), E2 concentration increases in the hypothalamus but not in the serum. **(B)** Like ethinyl estradiol (EE), DHED treatment reduces the rise of TST in the pharmacological model of hot flushes in OVX rats. Reproduced with modification from reference 96 (under a Creative Commons Attribution 4.0 International License).

In the present context, we showed that oral administration of DHED produced a significant E2 concentration in the hypothalamus (**Figure 4A**). This subsequently elicited a significant reduction of tail skin temperature (TST) rise representing hot flushes in the morphine-dependent model of this VMS after surgical menopause (ovariectomy) in rats (**Figure 4B**) and resulted in the restoration of estrogen deprivation-induced loss of diurnal rhythm in TST (106). In our experiments, the clinically used synthetic estrogen ethinyl estradiol (EE) was applied as positive control instead of E2 due to the poor oral bioavailability of the latter. It should be noted that VMS also affect prostate cancer patients undergoing androgen deprivation therapy (107). As seen in **Figure 2**, E2 is also formed in men from T; therefore, androgen deprivation robs the E2 source in men and results in the same hypoestrogenic brain as in women after menopause. However, conventional ET produces feminization (most prominently gynecomastia) in men, which erodes patients’ compliance because of physical and psychological discomfort arising from this effect (11). Therefore, confining E2 into the brain also is critical for these patients. Using orchiectomized rats in the same model of morphine-dependent hot flushes as for the female animals, we have also shown the beneficial TST lowering effect of the DHED-derived E2 (**Figure 3**) in male animals lacking gonadal E2 source (108).

Altogether, we believe DHED may have the potential to be the first efficacious brain-selective estrogen therapy with inherent safety (i.e., no significant liabilities in the periphery) to alleviate hot flushes and, thus, to improve health-related quality of life for millions suffering from VMS. Constraining E2’s action to the brain would also eliminate the need of a progestin for women with intact uterus. Observational and animal studies have revealed risks associated with progestins (109, 110). An additional benefit of our DHED approach is that for the first time E2’s central effects can be isolated from peripheral hormonal interference. This should ultimately lead to enhancing our understanding of E2’s central versus peripheral actions, as well as their contributions to the observed overall estrogenic effects on the body including the brain.

## 5 Conclusion

E2 is a master regulator hormone in the brain and its decline due to chronological or pathological aging in a female brings about a set of unpleasant symptoms, often negatively affecting the quality of life. Chiefly among them are hot flushes, the hallmark of (peri)menopause that most midlife women experience with various intensity and duration. While the physiology of hot flushes or that of VMS is not completely understood, it appears to be related to abnormal changes in the hypothalamus, particularly in its preoptic and anterior areas. Hot flushes start with the gradual decline in circulating E2 during perimenopause and may continue beyond. The hypoestrogenic brain also brings about anomalous changes in, among others, signaling linked to certain neurotransmitters/neuromodulators. Specifically, knowledge on NKB signaling has been shown recently as a translational target for alleviation of hot flushes without involving estrogens. Because of the close relationship between the offset of hot flushes and the waning circulating E2, systemic ET nevertheless seems to be the most effective treatment against moderate-to-intense hot flushes to “re-estrogenize” the brain. Current ETs however carry significant risks and contraindications owing to multiple factors acting in concert. One of the major caveats with current ETs is the unwanted systemic hormonal exposure with adverse consequences in a large set of patients, even if E2 is used instead of CEE. On the other hand, confining E2’s beneficial impact to the site of action to make the brain “estrogenic” again would eliminate this burden. In this regard, our non-invasive DHED prodrug approach may offer for the first time a realistic translational value in terms of defining a therapeutic landscape to remedy the E2-deficient brain safely and efficaciously, including the alleviation or prevention of VMS.

## Author contributions

KP: Conceptualization, Funding acquisition, Supervision, Writing – original draft. LP: Funding acquisition, Writing – review & editing.
